# Not all lipoprotein plays a crucial role in Alzheimer’s disease

**DOI:** 10.1002/alz.094718

**Published:** 2025-01-09

**Authors:** Dinghao An, Yun Xu

**Affiliations:** ^1^ Peking Union Medical College, Beijing China; ^2^ Affiliated Drum Tower Hospital of Medical School, Nanjing University, NANJING, Jiangsu China

## Abstract

**Background:**

Hyperlipoprotein cholesterolemia increases the risk of Alzheimer’s disease(AD). LDL is mainly responsible for the risk. However, lipoprotein have different densities, different particle sizes, and different compositions. We wanted to know how lipoprotein increases the risk of AD and whether it is related to its particle size and different compositions.

**Method:**

The GWAS summary data of the lipoprotein panel comes from Borges CM in 2020, data of AD is from Benjamin Woolf in 2022. We investigated the causal relationship between different‐density lipoprotein particles, their compositions, and AD by performing bidirectional Mendelian Randomization analysis. The study is divided into four parts. First, we performed correlation analyses of particle sizes of different‐density lipoprotein. Second, we performed correlation analyses of the concentration of particles of different particle sizes in different‐density lipoprotein. Third, we performed correlation analyses of the content of specific compositions in different‐density lipoprotein with different particle sizes, respectively. Fourth, we performed correlation analyses of specific compositions and specific ratios of different particle sizes in different‐density lipoprotein, respectively.

**Result:**

In the panel, we found that some factors could increase the risk of developing AD, which included Phospholipids in IDL(p = 0.008) Free cholesterol in LDL(p<0.001) Total lipids in LDL (p = 0.001) Cholesterol in small LDL(p = 0.001) Phospholipids in large LDL(p = 0.001) Free cholesterol in medium LDL(p<0.001) Cholesterol in medium VLDL(p = 0.003) Cholesteryl esters in medium VLDL(p = 0.003) Free cholesterol in medium VLDL(p = 0.012) Cholesterol to total lipids ratio in large HDL (p = 0.033) Phospholipids to total lipids ratio in medium LDL(p<0.001) Free cholesterol to total lipids ratio in small HDL(p = 0.009) Cholesteryl esters to total lipids ratio in small VLDL(p = 0.004) Free cholesterol to total lipids ratio in small VLDL(p<0.001) Cholesterol to total lipids ratio in very large VLDL(p = 0.013) Cholesteryl esters to total lipids ratio in very large VLDL(p = 0.010). On the contrary, Phospholipids in medium HDL(p = 0.035) could decrease the risk of AD. However, we did not find other factors that affect the risk of AD.

**Conclusion:**

We first found a genetic association between AD and different parts of lipoprotein, specifically about particle size and lipoprotein compositions. Therefore, not all lipoprotein can affect the risk of AD. The continuous advancement of electrophoresis technology allows for the stratification of different particle sizes of lipoprotein and compositions.

